# Opportunistic migration and habitat use of the giant mottled eel *Anguilla marmorata* (Teleostei: Elopomorpha)

**DOI:** 10.1038/s41598-018-24011-z

**Published:** 2018-04-04

**Authors:** Takaomi Arai, Naoko Chino

**Affiliations:** 1 Environmental and Life Sciences Programme, Faculty of Science, Universiti Brunei Darussalam, Jalan Tungku Link, Gadong, BE 1410 Brunei Darussalam; 2 Atmosphere and Ocean Research Institute, The University of Tokyo, 5-1-5, Kashiwanoha, Kashiwa, Chiba 277-8564 Japan

## Abstract

Freshwater eels have fascinated biologists for centuries due to the spectacular long-distance migrations between their freshwater habitats and their spawning areas far out in the ocean. Although freshwater eels originated in the Indonesian region, remarkably little is known about the life history of tropical freshwater eels. The diverse migratory patterns and habitat choice between marine and freshwater environments by the giant mottled eel *Anguilla marmorata* Quoy & Gaimard, 1824 were examined by analysing the otolith strontium (Sr) and calcium (Ca) concentrations collected in Asian waters. The wide range of otolith Sr:Ca ratios indicated that the habitat use of *A. marmorata* was opportunistic among fresh, brackish, and marine waters. The present study first confirmed the occurrence of marine-resident eels that have never migrated into a freshwater habitat in *A. marmorata*. *A. marmorata* may have the same behavioural plasticity as temperate and other tropical anguillid species regarding whether to enter freshwater or to remain in estuarine and marine environments. Freshwater eels migrate flexibly among freshwater, brackish water, and seawater environments and it is now evident that their movement into freshwater is not an obligate migratory pathway but should be defined as an opportunistic catadromy, with marine and estuarine residents as ecophenotypes.

## Introduction

Freshwater eels of the genus *Anguilla*, being catadromous^1^, migrate between freshwater growth habitats and offshore spawning areas. Nineteen species/subspecies of *Anguilla* have been reported world-wide, thirteen of which are known to occur in tropical regions^2,3^ that are globally distributed in temperate, tropical, and subtropical areas. In general, freshwater eels are divided into temperate and tropical eels, based on their major distribution and ecological properties^3^. The tropical species are thought to be more closely related to the ancestral form than their temperate counterparts. Thus, studying the life history and migration of tropical eels may provide some clues to understanding: the nature of primitive forms of catadromous migration in anguillid eels; and how the migration of the species became established.

The giant mottled eel *Anguilla marmorata* is a unique tropical freshwater eel that reaches large sizes of 2 m in length with a maximum weight of 21 kg^4^. This species has the widest geographic distribution of the 19 species/subspecies of freshwater eels^2^ and is found longitudinally from the east coast of Africa to the Marquesas Islands in the southeast Pacific Ocean and as far north as southern Japan^2^. This species was also found at the Palmyra Atoll in the central Pacific^5^ and even farther to the east in the Galapagos Islands^6^, which may indicate that it has an even wider geographic range than previously thought. Because of the wide geographic range of *A. marmorata*, which is separated by several major landmasses, it is clearly unlikely to comprise a single panmictic population such as temperate anguillid species found in one particular region of a single ocean basin^7–11^. Therefore, the ecological and biological characteristics such as reproduction, life history, migration and habitat use in *A. marmorata* seem to be different for each population.

Information regarding the life history characteristics of the tropical eels has been gradually accumulated^12–22^. Hatching was estimated to occur throughout most of the year with a constant age at recruitment^15^. Year-round recruitment of tropical glass eels to the river mouth^12,19^ follows year-round spawning^18,21^ and a stable recruitment age^15^. Such a life history strategy differs from that of the temperate eels, which have a limited spawning season followed by a limited period of recruitment. However, little information is available on the tropical eels regarding their life history and migration during the yellow and silver eel stages after recruitment to coastal waters as glass eels.

The migratory history of several species of freshwater eels have been studied using microchemical techniques that determine the ratio of strontium to calcium (Sr:Ca ratio) in their otoliths. The Sr:Ca ratio in the otoliths of freshwater eels differs according to the time they spend in fresh water versus sea water^23^. Studies on strontium incorporation into eel otoliths of *Anguilla japonica* showed that the Sr:Ca level in their otoliths strongly correlated with the salinity of the water and was little affected by other factors such as water temperature, food and physiological factors^24^. Recently, Arai and Chino^25^ found that the Ca and Sr contents and the resultant Sr:Ca ratios in the rearing water significantly increased with salinity in the giant mottled eel *Anguilla marmorata*. Thus, the Sr:Ca ratios of otoliths could help to determine whether individual eels actually enter fresh water at the elver stage and remain in a fresh water, estuarine or marine environment until the silver eel stage, or whether they move between different habitats with differing salinity regimes.

The objectives of this study were to accumulate ecological and biological information regarding the migration after recruitment to coastal waters of the giant mottled eel *Anguilla marmorata* collected in East Asia and Southeast Asia, as there has been little available information concerning its migration. Furthermore, we discuss the evolution and occurrence of the diverse migrations of the eels based on a comprehensive analysis of the knowledge from present and previous studies on their life history.

## Results

### Biological characteristics

The total length (TL) of *Anguilla marmorata* ranged from 342 to 1350 mm with a mean ± SD of 649 ± 229 mm (n = 151). The body weight (BW) ranged from 87 to 6000 g with a mean ± SD of 822 ± 1090 g (n = 151) (Table 1).

**Table 1:** Specimens of the tropical eel *Anguilla marmorata* collected from six localities in Indonesia, Japan and Vietnam waters used in the present study.

Sampling location	Sample size	Total Length (mm)		Body Weight (g)		Migration pattern estimated from otolith microchemistry
		Mean ± SD	Range	Mean ± SD	Range	
Poso, Indonesia	15	723 ± 282	494–1290	1350 ± 128	400–3950	freshwater/estuarine/marine residents
Amami Islands, Japan	51	550 ± 80.6	367–787	415 ± 241	87.2–1280	freshwater/estuarine residents
Bonin Islands, Japan	15	918 ± 189	754–1350	2208 ± 1520	485–6000	freshwater/estuarine residents
Quang Tri, Vietnam	12	581 ± 209	342–1030	724 ± 1010	90.4–3390	freshwater/estuarine residents
Quang Ngai, Vietnam	12	516 ± 275	355–1138	728 ± 1400	100–4320	freshwater/estuarine/marine residents
Binh Dinh, Vietnam	10	884 ± 159	671–1120	1990 ± 1010	980–3270	freshwater/estuarine/marine residents

### Migratory history

**Figure 1:**
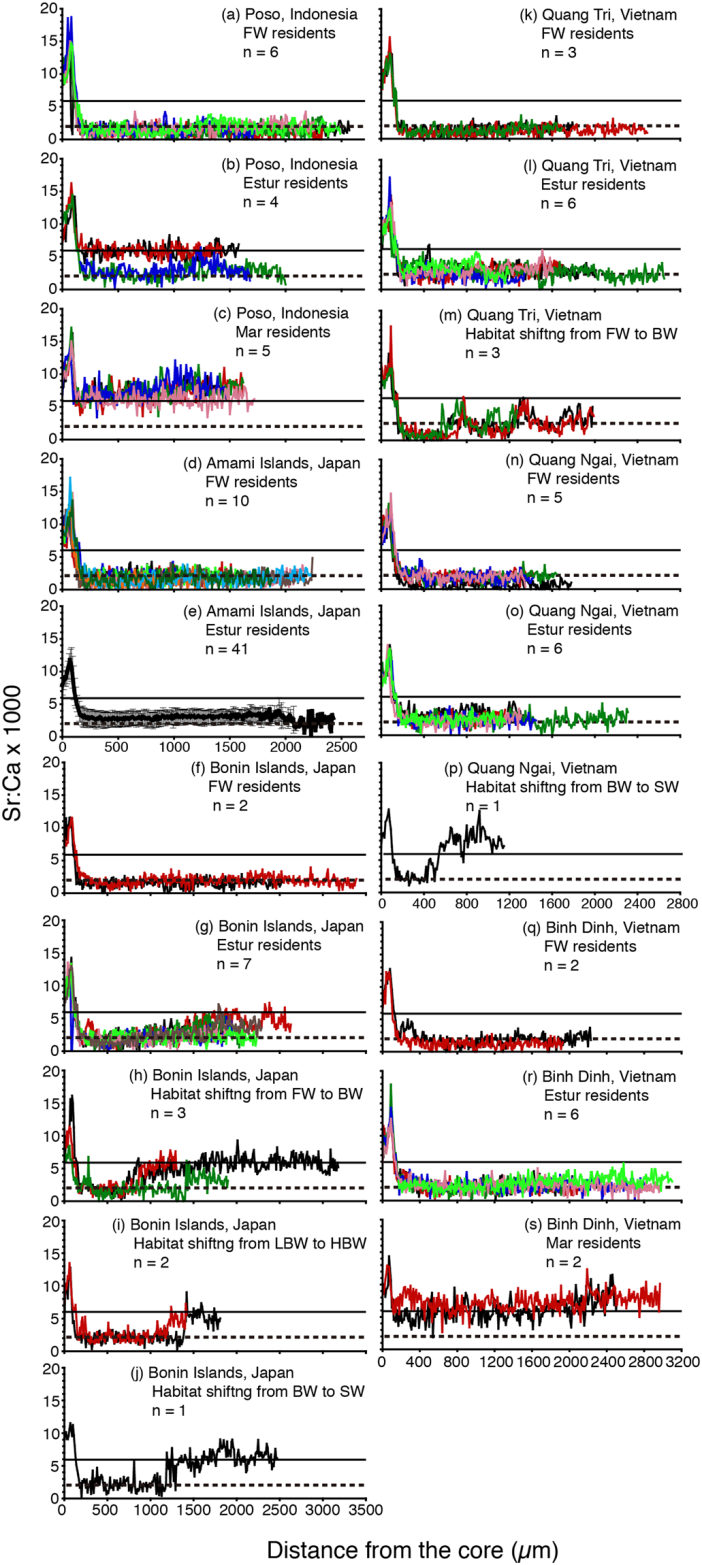
Migratory history of the giant mottled eel *Anguilla marmorata* as indicated by the temporal pattern of the Sr:Ca rations in their otoliths. Plots of otolith Sr:Ca ratios along a transect line from the core to the edge of the otolith for all specimens (115) of the giant mottled eel *Anguilla marmorata* in Indonesia, Japan and Vietnam. The solid line in each panel indicates the marine water life period (≥6.0 × 10^−3^ in Sr:Ca ratios), and the dotted line in each panel indicates the freshwater life period (<2.0 × 10^−3^ in Sr:Ca ratios). Numbers on upper right indicate fish number. Error bars indicate standard deviations. FW: freshwater, BW: brackish water, SW: sea water, LBW: lower brackish water, HBW: higher brackish water.

In all specimens examined, the otoliths had a central region of high Sr:Ca ratios that corresponded to the leptocephalus stage and showed lower Sr:Ca ratio levels after metamorphosis into the glass eel stage (Fig. 1). Each otolith had a peak between the otolith core and approximately 150 µm outward. The ratios in the otoliths of eels before the elver stage were similar among specimens, indicating that the migratory history was similar among specimens during the oceanic leptocephalus stage.

**Figure 2:**
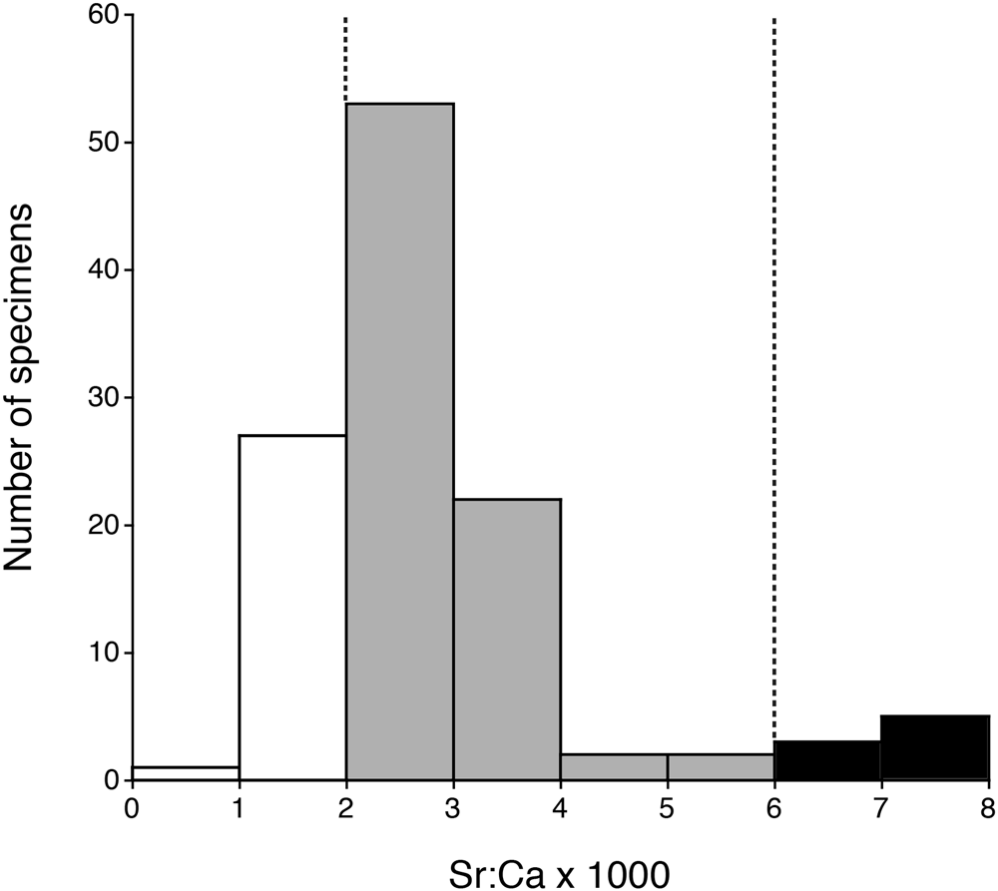
Frequency distribution of mean Sr:Ca ratio data outside the elver mark (150 µm in radius) of the giant mottled eel *Anguilla marmorata* in all fish collected from six localities in Indonesia, Japan, and Vietnam. The dotted line in each panel indicates either the freshwater life period (<2.0 × 10^−3^ in Sr:Ca ratios) or the marine water life period (≥6.0 × 10^−3^ in Sr:Ca ratios).

**Figure 3:**
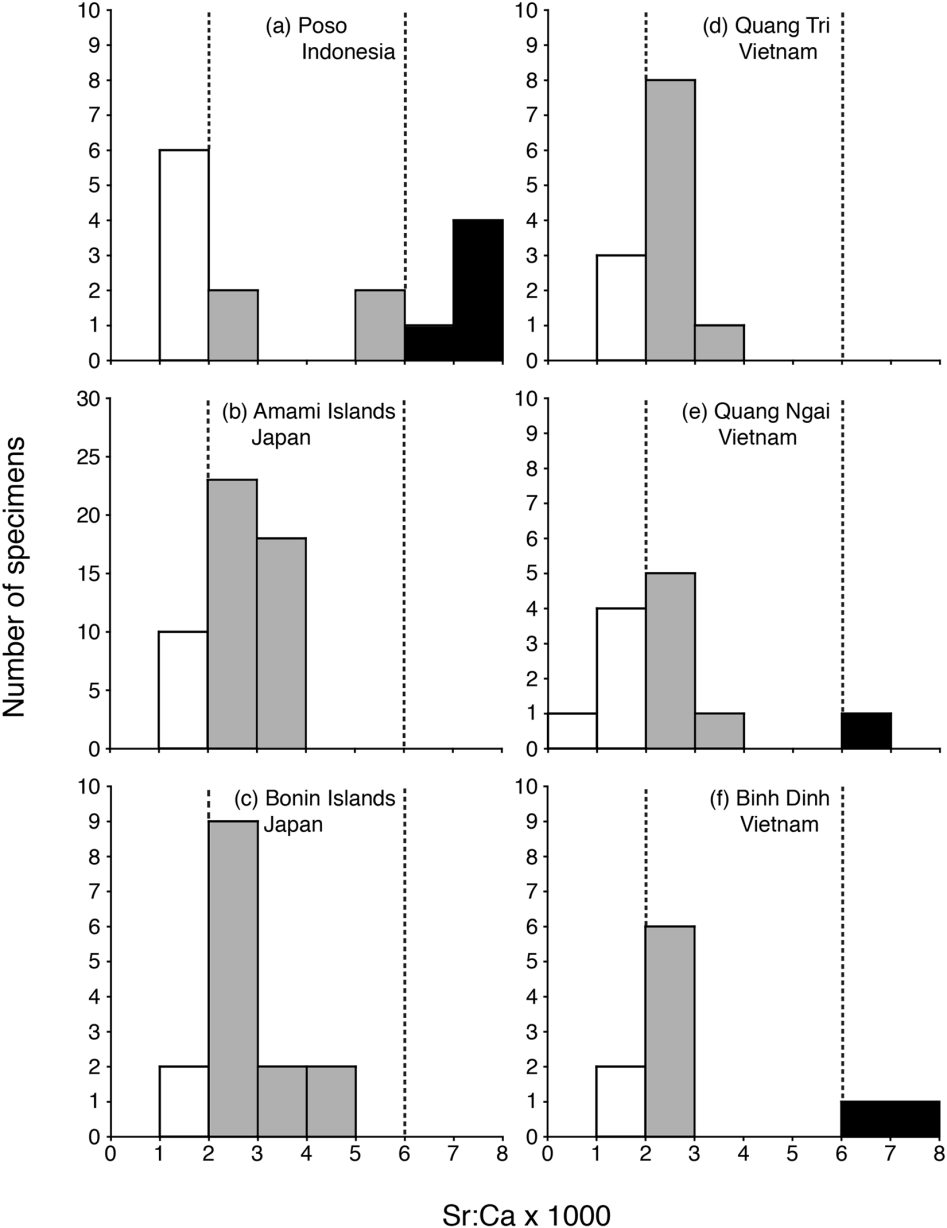
Frequency distribution of mean Sr:Ca ratio data outside the elver mark (150 µm in radius) of the giant mottled eel *Anguilla marmorata* collected at each sampling site. The dotted line in each panel indicates either the freshwater life period (<2.0 × 10^−3^ in Sr:Ca ratios) or the marine water life period (≥6.0 × 10^−3^ in Sr:Ca ratios).

Outside of the high Sr:Ca core, there were strong patterns in the Sr:Ca ratios of the eels’ otoliths. The patterns in the otolith Sr:Ca ratios from Poso River of Indonesia were classified into three constant types (Fig. 1a,b,c): (1) constantly low values of 1.23–1.77 × 10^−3^ (mean: 1.54 ± 0.20) (n = 6, Fig. 1a), (2) intermediate values of 2.56–5.95 × 10^−3^ (mean: 4.30 ± 1.80) (n = 4, Fig. 1b), and (3) relatively high values of 6.17–7.71 × 10^−3^ (mean: 7.17 ± 0.59) but larger than 6 × 10^−3^ with no movement into freshwater (n = 5, Fig. 1c). Significant differences in average Sr:Ca values between each type were found for all combinations (Kruskal-Wallis test, p < 0.05–0.0001).

The life history migratory patterns from Amami Islands in Japan were generally classified into two types (Fig. 1d,e): (1) constantly low values of 1.44–1.98 × 10^−3^ (mean: 1.74 ± 0.18) (n = 10, Fig. 1d), (2) intermediate values of 2.02–3.92 × 10^−3^ (mean: 2.91 ± 0.52) (n = 41, Fig. 1e). There were significant differences in average Sr:Ca values between the types (Mann-Whitney *U*-test, p < 0.0001). However, no eel showed relatively high Sr:Ca ratios more than 6 × 10^−3^ along the life history transect, which would indicate marine residence.

The eels from Bonin Islands of Japan showed unique and diverse migratory patterns classified into a total of five types with apparently two resident (Fig. 1f,g) and three migrant patterns (Fig. 1h,i,j). These two residents were (1) constantly low values of 1.51–1.92 × 10^−3^ (n = 2, Fig. 1f) and (2) intermediate values of 2.03–3.41 × 10^−3^ (mean: 2.48 ± 0.50) (n = 7, Fig. 1g). There were three groups of migrant types, with Sr:Ca ratios shifting between the inner and outer portion of the transect (Fig. 1h,i,j). In the first group (3), Sr:Ca ratios were low in the inner portion were low (range: 1.71–1.91 × 10^−3^; mean ± SD: 1.79 ± 0.10) and then increased to a middle range (range: 3.50–5.48 × 10^−3^; mean ± SD: 4.79 ± 1.12) (Fig. 1h, n = 3). The second group (4) showed Sr:Ca ratios that were lower intermediate level in the inner portion (2.09–2.31 × 10^−3^), then increased to a higher intermediate level but less than 6.0 × 10^−3^ in the outer portion of the transect (4.48–5.25 × 10^−3^) (Fig. 1i, n = 2). In the third group, Sr:Ca ratios were intermediate in the inner portion (2.11 × 10^−3^), then increased to a high level in the outer portion of the transect (6.14 × 10^−3^) (Fig. 1j, n = 1). For all migrant fishes, the transition occurred between 710 and 1400 µm from the otolith core. There were significant differences between the low and high Sr:Ca ratio phases for all migrant specimens (Mann-Whitney *U*-test p < 0.0001). Therefore, these transitions suggest that the migrant type specimens moved from lower salinity environments to higher salinity environments (Fig. 1h,i,j; n = 6).

The otolith Sr:Ca ratios along the life history transects of the eels from the Quang Tri in Vietnam showed two resident types and one migrant type (Fig. 1k,l,m). These two residents were (1) constantly low values of 1.24–1.55 × 10^−3^ (mean: 1.38 ± 0.16) (n = 3, Fig. 1k) and (2) intermediate values of 2.09–3.29 × 10^−3^ (mean: 2.81 ± 0.39) (n = 6, Fig. 1l). Significant differences were found in average Sr:Ca values between the types (Mann-Whitney *U*-test, p < 0.0005). The migrant type showed low values of 0.93–1.94 × 10^−3^ (mean ± SD: 1.33 ± 0.54) in the inner portion that then increased to a middle range of 2.57–3.30 × 10^−3^ (mean ± SD: 2.90 ± 0.37) at 610–950 µm from the otolith core (Fig. 1m, n = 3). There were significant differences between the low and high Sr:Ca ratio phases for those three specimens (Mann-Whitney *U*-test, p < 0.0001).

The eels in the Quang Ngai in Vietnam showed two resident types and one migrant type (Fig. 1n,o,p). These two residents were (1) constantly low values of 0.93–1.98 × 10^−3^ (mean: 1.73 ± 0.45) (n = 5, Fig. 1n) and (2) intermediate values of 2.28–3.39 × 10^−3^ (mean: 2.56 ± 0.42) (n = 6, Fig. 1o). There were significant differences in average Sr:Ca values between the types (Mann-Whitney *U*-test, p < 0.05).The migrant type showed Sr:Ca ratios that were intermediate in the inner portion (2.21 × 10^−3^), then increased to a high level in the outer portion of the transect (7.57 × 10^−3^) at 430 µm from the otolith core (Fig. 1p, n = 1). There were significant differences between the low and high Sr:Ca ratio phases for those three specimens (Mann-Whitney *U*-test, p < 0.0001).

The patterns in the otolith Sr:Ca ratios from the Binh Dinh in Vietnam were classified into three constant types (Fig. 1q,r,s): (1) constantly low values of 1.28–1.95 × 10^−3^ (n = 2, Fig. 1q), (2) intermediate values of 2.02–2.83 × 10^−3^ (mean: 2.34 ± 0.27) (n = 6, Fig. 1r), and (3) relatively high values of 6.15–7.34 × 10^−3^ (n = 2, Fig. 1s).

In *Anguilla marmorata* collected from six localities, the numbers of freshwater, estuarine and marine residents were 28, 79 and 8, respectively (Fig. 2). The estuarine resident eels were further classified into four types: (1) constant (70 specimens), (2) habitat shifting from freshwater to brackish water (6 specimens), (3) habitat shifting from lower brackish water to higher brackish water (2 specimens) and (4) habitat shifting from brackish water to sea water (1 specimen) (Fig. 1). The marine resident eels were further classified into two types: (1) constant (7 specimens), (2) habitat shifting from freshwater to seawater (1 specimen) (Fig. 1).

### Habitat use

The mean Sr:Ca ratio value beyond 150 μm from the otolith core in each eel ranged from 0.93 to 7.71 × 10^−3^ (Fig. 2). However, the mean values were different at each site. The mean Sr:Ca ratio values at Poso River of Indonesia ranged from 1.23 to 7.71 × 10^−3^ with a mean ± SD of 4.15 × 10^−3^ ± 2.65 × 10^−3^. Three migratory types, freshwater, estuarine and marine residents were found in the region (Fig. 3a). In Amami and Bonin islands of Japan, the mean Sr:Ca ratio values ranged from 1.44 to 3.92 × 10^−3^ with a mean ± SD of 2.68 × 10^−3^ ± 0.66 × 10^−3^ and from 1.51 to 4.82 × 10^−3^ with a mean ± SD of 2.75 × 10^−3^ ± 0.92 × 10^−3^, respectively. There were two migratory types, freshwater and estuarine residents in those islands (Fig. 3b,c). These values in the Quang Tri, Quang Ngai and Binh Dinh of Vietnam ranged from 1.24 to 3.29 × 10^−3^ with a mean ± SD of 2.33 × 10^−3^ ± 0.67 × 10^−3^, from 0.93 to 6.14 × 10^−3^ with a mean ± SD of 2.51 × 10^−3^ ± 1.28 × 10^−3^ and from 1.28 to 7.34 × 10^−3^ with a mean ± SD of 3.07 × 10^−3^ ± 1.99 × 10^−3^, respectively. In the Quang Ngai and Binh Dinh, freshwater, estuarine and marine residents were found (Fig. 3e,f), while freshwater and estuarine residents were found in the Quang Tri (Fig. 3d). The percentages of each migratory type, freshwater residents, estuarine residents and marine residents, in Poso in Indonesia, Amami and Bonin islands of Japan and Quang Tri, Quang Ngai and Binh Dinh of Vietnam were 40%, 26.7% and 33.3%; 19.6%, 80.4% and 0%; 13.3%, 86.7% and 0%; 25%, 75% and 0%; 41.7%, 50% and 8.3% and 20%, 60% and 20%, respectively.

The wide range of otolith Sr:Ca ratios indicated that the habitat use of *Anguilla marmorata* was variable after their recruitment to coastal waters as glass eels in Indonesia, Japan and Vietnam waters (Fig. 2). The overall percentage of each migratory type, freshwater residents, estuarine residents and marine residents, for all samples was 24.3%, 68.7% and 7%. Freshwater residence, which is the typical catadromous migratory pattern of freshwater eel migration, was lower, while estuarine residence was dominant among the three migratory types in the species.

## Discussion

The occurrence of marine resident eels that have never migrated into freshwater habitat was first confirmed in the giant mottled eel *Anguilla marmorata* (Figs 2 and 3), although marine residence has been found in other species and localities in coastal waters for both temperate and tropical eels^23,26–42^. In contrast, all *A. marmorata* and *A. celebesensis* eels in the freshwater Lake Poso of Indonesia showed constantly low Sr:Ca values (<2.0 × 10^−3^, Table 2), which could thus be considered as a useful indicator of freshwater residence^41^. This lake is the third largest freshwater lake in Indonesia and the lake is connected to the sea by a single river, the Poso River, which drains the water from an elevation of 512 m past a waterfall and down to the sea along a 40-km stretch. A number of *A. marmorata* and *A. celebesensis* eels were collected in the uppermost freshwater lake and live sympatrically in the lake^20^. *Anguilla marmorata* is thought to be an ancient tropical eel that has a preference for freshwater in the growth phase, which causes the eel to migrate upstream^36^. In Taiwan, *A. marmorata* was mainly freshwater-oriented and apparently avoided residence in a brackish water environment, while *A. japonica* had a more phenotypic plasticity than *A. marmorata*^36^. These two eel species were though to have interspecific resource competition by allopatric rather than sympatric distribution in Taiwan^36^. Therefore, the habitat use by two eel species between Lake Poso of Indonesia and Taiwan might be different. The difference in migratory behavior and habitat use between species in Taiwan is hypothesized to be because that the brackish water habitat was overwhelmingly dominated by *A. japonica*^36^. *A. marmorata* was expelled to the upper reaches, where the habitats were less productive and unstable^36^. In this study, however, it was found that estuarine residents and marine residents were dominant constituting 58% to 87% in all sites, while freshwater residents ranged from 13% to 42%. Therefore, the habitat preference of *A. marmorata* would not be a result of its phylogenetic traits but by its environmental adaptations to various habitats and salinities. Environmental factors as well as the interspecific interactions might influence the habitat use of fish. The specific differences in habitat use should be carefully interpreted because the difference might not be a simple interaction among competitors^43^. To understand the details of the migratory behavior and habitat use of *A. marmorata*, further otolith microchemical analyses should be undertaken collecting from various environmental (salinity) habitats.

**Table 2:** Range of otolith Sr:Ca ratios outside the elver mark in *Anguilla marmorata*.

Country	Sr/Ca ratios	Reference
Poso, Indonesia	1.2–7.6 × 10^−3^	This study
Amami Islands, Japan	1.4–3.9 × 10^−3^	This study
Bonin Islands, Japan	1.5–4.8 × 10^−3^	This study
Quang Tri, Vietnam	1.2–3.3 × 10^−3^	This study
Quang Ngai, Vietnam	0.9–6.1 × 10^−3^	This study
Binh Dinh, Vietnam	1.3–7.3 × 10^−3^	This study
Indonesia (Lake Poso)	0.8–0.9 × 10^−3^	Chino and Arai^41^
Japan	1.1–3.2 × 10^−3^	Chino and Arai^39^
Philippines^※^	1.5 ± 0.9 × 10^−3^	Briones *et al*.^44^
South Africa	2.7–2.8 × 10^−3^	Lin *et al*.^42^
Taiwan	1.6–2.2 × 10^−3^	Shiao *et al*.^36^
Vietnam	1.7–5.7 × 10^−3^	Arai *et al*.^33^

^※^Mean ± SD.

The habitat preference in terms of salinity environment is also though to depend on whether there are multiple species of anguillid eels in the habitat or a single species^23,33^. In the Philippines and Taiwan, *A. marmorata* tended to live in freshwater environments^36,44^, while all localities in Indonesia, Japan and Vietnam examined in the present study showed various migratory patterns (Table 1, Fig. 3). There were *A. bicolor pacifica* and *A. marmorata* in the Philippines and *A. marmorata* and *A. japonica* in Taiwan, while only *A. marmorata* was observed in the Amami and Bonin islands of Japan. In those areas, *A. marmorata* could live in every environment with no interspecific competition, and thus estuarine-resident eels may be more abundant there than in the Philippines and Taiwan. In the Poso River of Indonesia, *A. celebesensis* was found in Lake Poso, which is the uppermost area in the Poso River^20,41^. However, we did not find *A. celebesensis* downstream in the Poso River. Therefore, habitat segregation might occur between *A. marmorata* and *A. celebesensis* in the river system. In New Zealand, short-finned eels *Anguilla australis* and long-finned eels *Anguilla dieffenbachii* were found to occur with the former usually predominating in lowlands but not usually predominating as far upstream as long-finned eels^45^. In Vietnam, the previous study did not find marine resident *A. marmorata* eels in Ba River, which is close to the current study sites of Quan Ngai and Binh Dinh. There the occurrence of *A. bicolor pacifica* and *A. marmorata* was observed in Vietnam waters^33^. Most *A. marmorata* were estuarine residents (93.3%), while 6.7% were freshwater residents; no *A. marmorata* were marine residents in Ba River in Vietnam^33^. In the present study, the dominance of estuarine residents in *A. marmorata* was found at three sites ranging from 50% to 75% in Vietnam. In Vietnam, most *A. bicolor pacifica* were also estuarine residents (88.9%), but 11.1% were marine residents in the river; no *A. bicolor pacifica* were freshwater residents^33^. Therefore, *A. bicolor pacifica* might prefer to live in higher salinity environments than *A. marmorata* in Vietnam waters. These results all lead to the conclusion that *A. marmorata* might be able to take advantage of various salinity environments during their lives in relation to the occurrence of either multiple or single species of anguillid eels.

**Figure 4:**
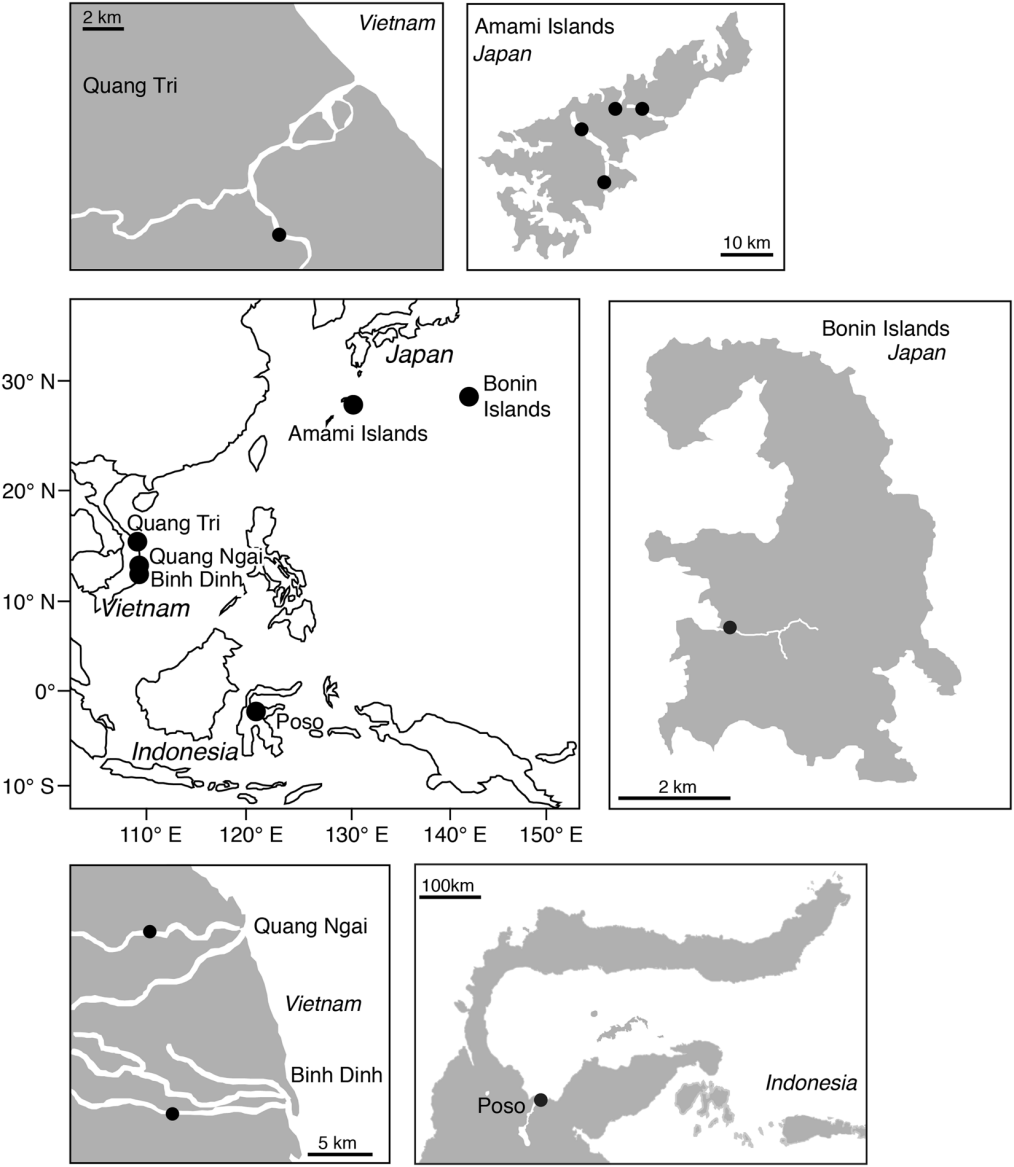
Locations where the giant mottled eel *Anguilla marmorata* were collected. Map of sampling locations for the giant mottled eel *Anguilla marmorata* in Indonesia, Japan and Vietnam. The map was traced by the author used the Adobe Illustrator CS6 referring Google Maps 2017 (Map data ©2017 Google; https://maps.google.com/).

Other possible explanations for the difference in migratory behaviour and habitat use might be differences among regions in habitat environment such as carrying capacity, current velocity, bottom material and inclination pitch. In New Zealand, habitat segregation occurs with physical parameters in the habitat environments^46^. Long-finned eels *Anguilla dieffenbachii* tended to associate with faster water velocities and larger substrates for riffles, while shortfinned eels *Anguilla australis* occupied slower marginal habitats. The rivers in Amami and Bonin islands of Japan are much smaller than those of Indonesia and Vietnam (Fig. 4); although, we did not conduct a detailed survey of river characteristics in this study. Most areas of those rivers in Japan were influenced by rising tide, with freshwater areas being much more limited compared to other sites in Indonesia and Vietnam, and therefore the migratory types of *A. marmorata* in those islands were estuarine residents constituting more than 80%. The migratory behaviour of anguillid eels would have phenotypic plasticity in each habitat in response to differences in physical habitat conditions after recruitment. Further study should be undertaken in the field with detailed migratory history analyses along the river system to elucidate the valid mechanisms of habitat use and habitat segregation in each species.

Another significant result was the finding of an intermediate constant type of eel migration (estuarine resident) and switching types of migration (Fig. 1). A predominance of eels that move once or several times between habitats of different salinity has been reported for a number of anguillid eel species examined^23^. The results have demonstrated that eels found in coastal and estuarine habitats can be resident in these areas and may move back and forth between freshwater and seawater. These findings indicate that the tropical eel *A. marmorata* has a flexible migration strategy with a high degree of behavioural plasticity and an ability to utilize the full range of salinity similar to that of the tropical eels *A. bicolor pacifica*^33^ and *A. bicolor bicolor*^40,41^ and the temperate eels *A. japonica*^26–28,31,32,34,35,38^, *A. anguilla*^30^, *A. rostrata*^37^, *A. australis* and *A. dieffenbachii*^29^. The freshwater eels migrate flexibly among freshwater, brackish water, and seawater environments, and it is now evident that their movement into freshwater is not an obligate migratory pathway and should be defined as an opportunistic catadromy, with ocean and estuarine residents as ecophenotypes.

Higher proportions of estuarine- and marine-resident eels have been hypothesized to live at the higher latitudes than in subtropical to tropical regions^47^. The freshwater eels that recruit at low latitudes migrate upstream into freshwater habitats of higher productivity for growth before returning to the ocean for breeding. Therefore, a latitudinal cline might be predicted in which marine resident freshwater eels would occur more frequently at higher latitudes where the productivity of the fresh water habitat is lower compared to the ocean. However, the broad ranges of otolith Sr:Ca ratios found in tropical eels in the present and previous^33,39–41^ studies suggest that these species have flexible migratory behaviour in ambient waters. The habitat preference of these tropical eels during the growth phase was the same as that of the temperate eels *A. anguilla*, *A. rostrata*, *A. japonica*, *A. australis* and *A. dieffenbachii*^26–32,36–38^. Therefore, the present and previous results regarding the habitat uses of tropical eels did not support the hypothesis. The ability of anguillid eels to reside in environments of varying salinity and to do so without a latitudinal cline may be a common feature of both tropical and temperate species. Freshwater eels are hypothesized to have originated from a marine ancestor^48^, and all anguilliform fishes except freshwater eels are marine species; thus the marine breeding habits of freshwater eels are considered a conservative trait. This suggests the hypothesis that a number of both tropical and temperate species of catadromous eels never lost the ability to reside in marine and estuarine habitats during the juvenile growth phase, but it is unknown whether this is due to a remnant genetic trait that determines whether or not an individual will enter freshwater or if it is simply due to behavioral plasticity that enables each species to use the maximum range of habitats.

## Methods

### Eels

A total of 115 specimens of the giant mottled eel *Anguilla marmorata* were collected from one site in Indonesia (15 specimens), two sites in Japan (66 specimens) and three sites in Vietnam (34 specimens) waters through traps, hooks and lines (Fig. 4, Table 1). No specific permissions were required for these locations/activities, as the eel species involved is not endangered or protected and the collection area did not require permits to collect these animals. Our protocol was in accordance with a guide for animal experimentation at Universiti Brunei Darussalam (UBD) and fish-handling approval was granted by the animal experiment committee of UBD.

In Indonesia, eels were collected in the tidal zone of Poso River, central Sulawesi Island, Indonesia on 25 July 2009 (Fig. 4). In Japan, 51 eels were collected in the tidal zone of four rivers in the Amami Islands of southern Japan in the East China Sea between August 2007 and December 2008 (Fig. 4). A total of 15 specimens were collected by fishing and harpoon in the Yatsuse River of the Bonin Islands, Japan between September 2008 and March 2009 (Fig. 1). The sampling sites were influenced by the rising tide (0.0–7.8‰ in salinity). Eels were collected from three areas, Quang Tri (12 specimens) Quang Ngai (12 specimens) and Binh (10 specimens) provinces, in the central part of Vietnam between April 2007 and March 2008 (Fig. 4). All sites were influenced by rising tide. The TL and BW of each eel was measured (Table 1).

### Otolith preparation and microchemical analysis

Sagittal otoliths were extracted from each fish, embedded in epoxy resin (Struers, Epofix) and mounted on glass slides. The otoliths were then ground and polished as described by Arai *et al*. (2013). They were then cleaned in an ultrasonic bath and rinsed with deionized water prior to examination. For electron microprobe analyses, all otoliths were Pt-Pd-coated by a high vacuum evaporator. All otoliths were used for “life-history transect” analysis of Sr and Ca concentrations, which were measured along a line down the longest axis of each otolith from the core to the edge using a wavelength dispersive X-ray electron microprobe (JEOL JXA-8900R) as described in Chino and Arai^39–41^. Wollastonite (CaSiO_3_) and tausonite (SrTiO_3_) were used as standards. The accelerating voltage and beam current were 15 kV and 1.2 × 10^−8^ A, respectively. The electron beam was focused on a point 10 µm in diameter, with measurements spaced at 10 µm intervals.

We calculated the average Sr:Ca ratio for the values outside the elver mark, and we categorized the eel habitats into “marine” (Sr:Ca ≥ 6.0 × 10^−3^), “estuarine” (2.0 × 10^−3^ ≤ Sr:Ca < 6.0 × 10^−3^) and “freshwater” (Sr:Ca < 2.0 × 10^−3^) according to the criteria for tropical eels including *Anguilla marmorata* by Chino and Arai^39,40^ and Arai and Chino^23,25^.

### Statistical analysis

The differences in the average Sr:Ca ratio for the values outside the elver mark among migratory types were examined through a Kruskal-Wallis test. Consequently, post hoc Mann-Whitney-*U* tests were employed for between-species comparisons. Differences in the average Sr:Ca ratio for the values outside the elver mark between migratory types and between the low and high phases were examined through a Mann-Whitney-*U* test.
